# Correction to: SALP, a new single-stranded DNA library preparation method especially useful for the high-throughput characterization of chromatin openness states

**DOI:** 10.1186/s12864-018-4688-8

**Published:** 2018-05-04

**Authors:** Jian Wu, Wei Dai, Lin Wu, Jinke Wang

**Affiliations:** State Key Laboratory of Bioelectronics, Southeast University, Sipailou 2, Nanjing, 210096 China

## Correction

After publication of the original article [1] the authors noted that the additional files had not been uploaded correctly.

While the legends are correctly cited, the figures themselves are incorrect.

The correct order of additional files follows below:

**Table:** 

**Original Additional Files**	**Correct Additional Files**
Additional File 1	Additional File 10
Additional File 2	Additional File 11
Additional File 3	Additional File 1
Additional File 4	Additional File 2
Additional File 5	Additional File 3
Additional File 6	Additional File 4
Additional File 7	Additional File 5
Additional File 8	Additional File 6
Additional File 9	Additional File 7
Additional File 10	Additional File 8
Additional File 11	Additional File 9

A full list of the correct corresponding files and their legends are included with this Correction.

The publisher apologises for this error.

## Additional files

Additional file 1: **Table S1.** Oligonucleotides used as adaptors and PCR primers. (DOCX 15 kb)

Additional file 2: **Table S2.** Barcodes on Barcoded Tn5 adaptors for labeling different cell samples. (DOCX 14 kb)

Additional file 3: **Figure S1.** Validation of SALP method. (DOCX 309 kb)

Additional file 4: Cloning sequencing. (DOCX 21 kb)

Additional file 5: **Figure S2.** The structure of SALP library. (DOCX 68 kb)

Additional file 6: **Figure S3.** Verification of the ligation efficiency of SSA adaptors. (DOCX 187 kb)

Additional file 7: **Table S3.** Reads from a lane of Illumina Hiseq X Ten sequencing. (DOCX 15 kb)

Additional file 8: **Figure S4.** Comparison of fold enrichment of two types of GM12878 SALP-seq peaks. (DOCX 45 kb)

Additional file 9: **Figure S5.** Construction of NGS library of gDNAs sheared by sonication and restriction endonuclease digestion with SALP method. (DOCX 295 kb)

Additional file 10: **Figure S6.** Comparison of the distribution of Hind III digestion library reads density and Hind III restriction sites through the whole genome. (DOCX 444 kb)

Additional file 11: **Figure S7.** Reads distribution of sonication library. (DOCX 395 kb)
