# Supplementary material for: SALP, a new single-stranded DNA library preparation method especially useful for the high-throughput characterization of chromatin openness states
Source: BMC Genomics. 2018 Feb 13;19:143. doi: 10.1186/s12864-018-4530-3 (PMC5811972; doi:10.1186/s12864-018-4530-3)
Supplement: Supplementary file 1 — Table S1. Oligonucleotides used as adaptors and PCR primers. (DOCX 444 kb) [file 12864_2018_4530_MOESM10_ESM.docx]

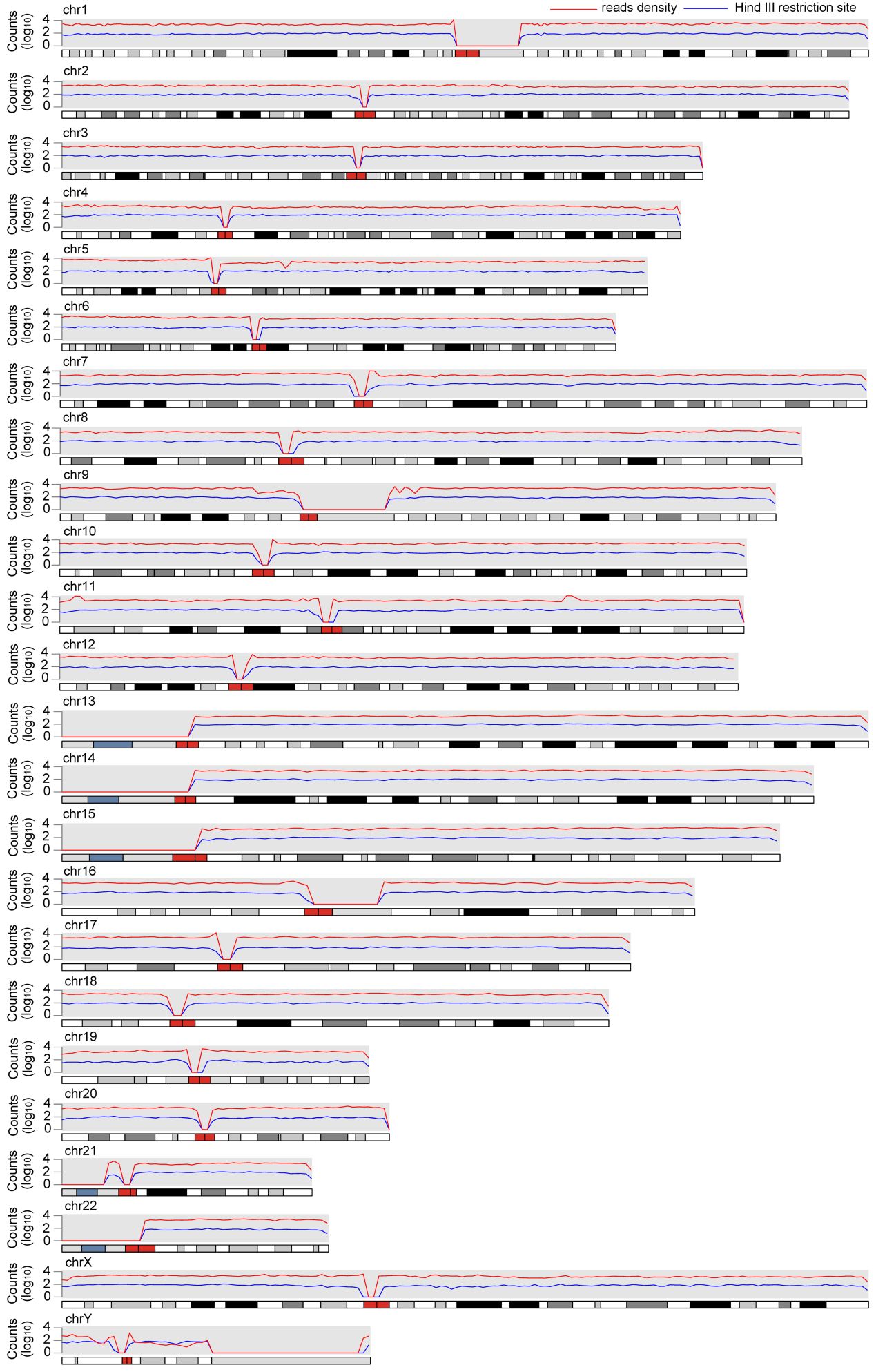


**Fig. S6. Comparison of the distribution of Hind III digestion library reads density and Hind III restriction sites through the whole genome.** The Hind III digestion library reads density and Hind III restriction sites density were calculated through the whole genome scale with 1M window.
