# Supplementary material for: SALP, a new single-stranded DNA library preparation method especially useful for the high-throughput characterization of chromatin openness states
Source: BMC Genomics. 2018 Feb 13;19:143. doi: 10.1186/s12864-018-4530-3 (PMC5811972; doi:10.1186/s12864-018-4530-3)
Supplement: Supplementary file 2 — Table S2. Barcodes on Barcoded Tn5 adaptors for labeling different cell samples. (DOCX 395 kb) [file 12864_2018_4530_MOESM11_ESM.docx]

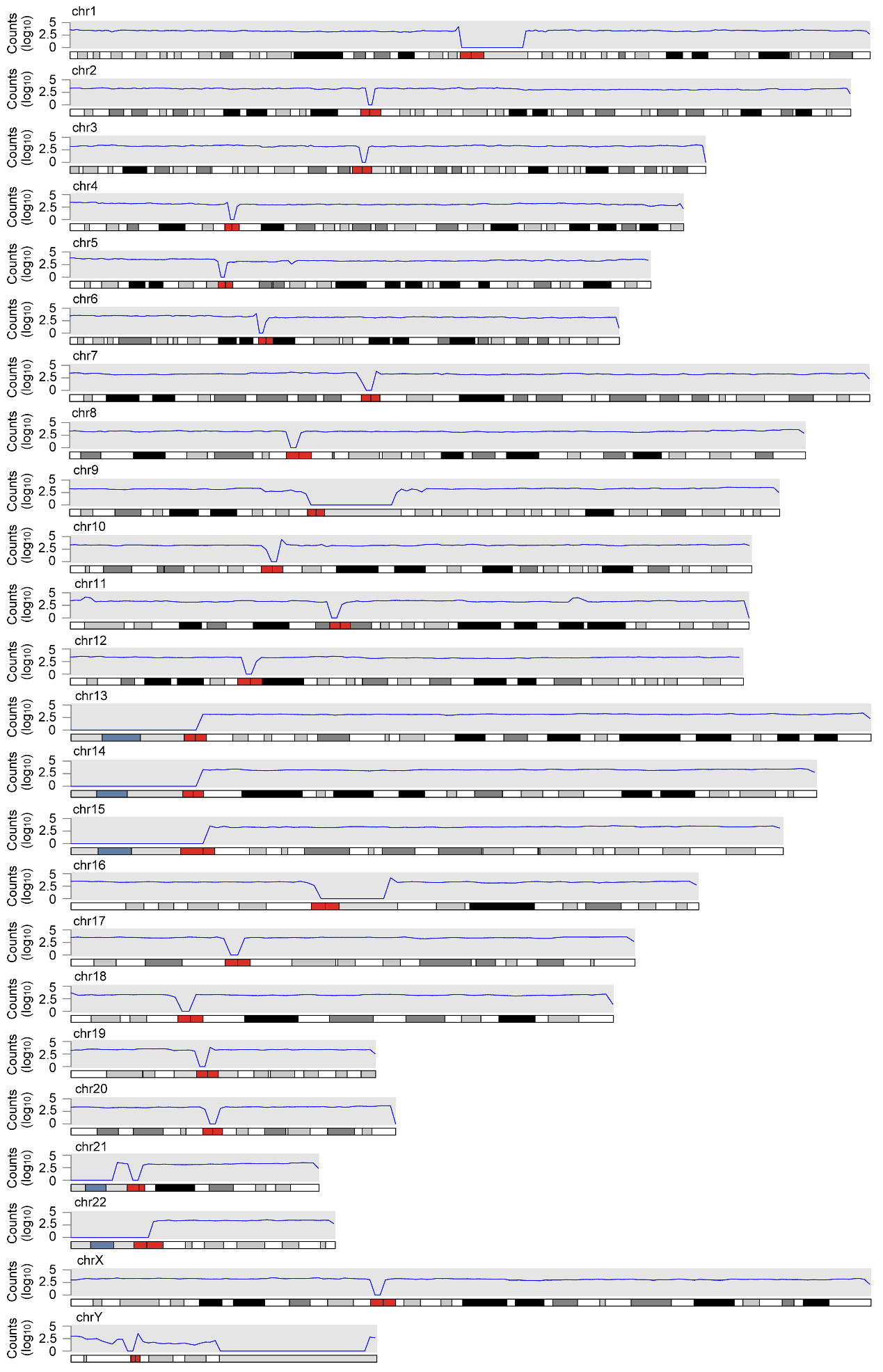


**Fig. S7. Reads distribution of sonication library.** The reads dense of sonication library in whole genome scale were calculated with 1 M window.
