# Supplementary material for: SALP, a new single-stranded DNA library preparation method especially useful for the high-throughput characterization of chromatin openness states
Source: BMC Genomics. 2018 Feb 13;19:143. doi: 10.1186/s12864-018-4530-3 (PMC5811972; doi:10.1186/s12864-018-4530-3)
Supplement: Supplementary file 3 — Figure S1. Validation of SALP method. (DOCX 15 kb) [file 12864_2018_4530_MOESM1_ESM.docx]

**Table S1 Oligonucleotides used as adaptors and PCR primers**

| **Barcoded Tn5 Adaptors** | | |
| --- | --- | --- |
| Name | Sequence (5' > 3') | Usage |
| Barcode1 | GACTGGAGTTCAGACGTGTGCTCTTCCGATCTTAGCTT  AGATGTGTATAAGAGACAG | to anneal with ME oligo |
| Barcode2 | GACTGGAGTTCAGACGTGTGCTCTTCCGATCTCTTGTA  AGATGTGTATAAGAGACAG |  |
| Barcode3 | GACTGGAGTTCAGACGTGTGCTCTTCCGATCTGCCAAT  AGATGTGTATAAGAGACAG |  |
| Barcode4 | GACTGGAGTTCAGACGTGTGCTCTTCCGATCTTGACCA  AGATGTGTATAAGAGACAG |  |
| Barcode5 | GACTGGAGTTCAGACGTGTGCTCTTCCGATCTATCACG  AGATGTGTATAAGAGACAG |  |
| Barcode6 | GACTGGAGTTCAGACGTGTGCTCTTCCGATCTACTTGA  AGATGTGTATAAGAGACAG |  |
| Barcode7 | GACTGGAGTTCAGACGTGTGCTCTTCCGATCTCGATGT  AGATGTGTATAAGAGACAG |  |
| Barcode8 | GACTGGAGTTCAGACGTGTGCTCTTCCGATCTACAGTG  AGATGTGTATAAGAGACAG |  |
| Barcode9 | GACTGGAGTTCAGACGTGTGCTCTTCCGATCTCAGATC  AGATGTGTATAAGAGACAG |  |
| ME oligo | [phos]CTGTCTCTTATACACATCT | to anneal with Barcode1‒9 |
|  |  |  |
| **Single strand adaptors (SSA)** | | |
| Name | Sequence (5' > 3') | Usage |
| SSA-PN | [phos]-AGATCGGAAGAGCGTCGTGTAGGGAAAGAGTGT-[NH2] | to anneal with SSA-PNrev |
| SSA-PNrev-1N | ACACTCTTTCCCTACACGACGCTCTTCCGATCTN | to anneal with SSA-PN |
| SSA-PNrev-2N | ACACTCTTTCCCTACACGACGCTCTTCCGATCTNN | to anneal with SSA-PN |
| SSA-PNrev-3N | ACACTCTTTCCCTACACGACGCTCTTCCGATCTNNN | to anneal with SSA-PN |
| SSA-PNrev-4N | ACACTCTTTCCCTACACGACGCTCTTCCGATCTNNNN | to anneal with SSA-PN |
|  |  |  |
| **T adaptors** |  |  |
| Name | Sequence (5' > 3') | Usage |
| TOA | GACTGGAGTTCAGACGTGTGCTCTTCCGATCTT | to anneal with TOArev |
| TOArev | [phos]-AGATCGGAAGAGCACACGTCTGAACTCCAGTC-[NH_2_] | to anneal with TOA |
|  |  |  |
| **PCR Primers for preparation of Illumina compatible library** | | |
| Name | Sequence (5' > 3') | Usage |
| NEBNext Universal PCR Primer | AATGATACGGCGACCACCGAGATCTACACTCTTTCCCTACACGACGCTCTTCCGATC*T | to amplify illumina compatible library;  3'phosphorothioate bond |
| NEBNext Index 13 Primer | CAAGCAGAAGACGGCATACGAGATTGTTGACTGTGACTGGAGTTCAGACGTGTGCTCTTCCGATC*T |  |
| NEBNext Index 14 Primer | CAAGCAGAAGACGGCATACGAGATACGGAACTGTGACTGGAGTTCAGACGTGTGCTCTTCCGATC*T |  |
| NEBNext Index 15 Primer | CAAGCAGAAGACGGCATACGAGATTCTGACATGTGACTGGAGTTCAGACGTGTGCTCTTCCGATC*T |  |

Note: 5'-P is for ligation; 3'-NH_2_ prevents undesired ligation.
