# Supplementary material for: SALP, a new single-stranded DNA library preparation method especially useful for the high-throughput characterization of chromatin openness states
Source: BMC Genomics. 2018 Feb 13;19:143. doi: 10.1186/s12864-018-4530-3 (PMC5811972; doi:10.1186/s12864-018-4530-3)
Supplement: Supplementary file 4 — Cloning sequencing. (DOCX 14 kb) [file 12864_2018_4530_MOESM2_ESM.docx]

**Table S2. Barcodes on Barcoded Tn5 adaptors for labeling different cell samples.**

| **Sample** | Number of Barcode | **Barcode** |
| --- | --- | --- |
| GM12878 10^5^ cells | Barcode1 | TAGCTT |
| HepG2 10^5^ cells | Barcode2 | CTTGTA |
| HeLa 10^5^ cells | Barcode3 | GCCAAT |
| 293T 10^5^ cells | Barcode4 | TGACCA |
| HepG2 5×10^4^ cells | Barcode5 | ATCACG |
| HepG2 1×10^4^ cells | Barcode6 | ACTTGA |
| HepG2 5×10^3^ cells | Barcode7 | CGATGT |
| HepG2 2.5×10^3^ cells | Barcode8 | ACAGTG |
| HepG2 5×10^2^ cells | Barcode9 | CAGATC |
