# Supplementary material for: SALP, a new single-stranded DNA library preparation method especially useful for the high-throughput characterization of chromatin openness states
Source: BMC Genomics. 2018 Feb 13;19:143. doi: 10.1186/s12864-018-4530-3 (PMC5811972; doi:10.1186/s12864-018-4530-3)
Supplement: Supplementary file 5 — Figure S2. The structure of SALP library. (DOCX 309 kb) [file 12864_2018_4530_MOESM3_ESM.docx]

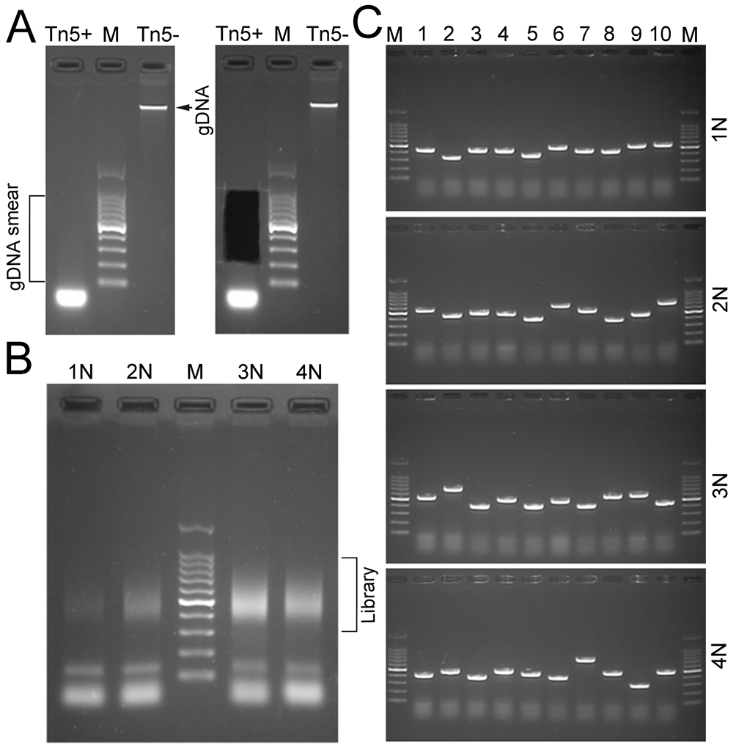


**Supplementary Fig.1. Validation of SALP method.** (**A**) The HepG2 genomic DNA (gDNA) tagmented by Tn5 transposome. The tagmented gDNA showed as smear in comparison with the input DNA. DNA smear was cut and purified. (**B**) Illumina compatible libraries were constructed using different SSAs with different numbers of overhanging bases at 3ʹ end. The SSA with 3N overhang was adopted for its high-ligation efficiency. 1N‒4N: SSAs with different numbers of random bases. (**C**) Clone sequencing was performed with the libraries constructed with 4 different SSAs to check the libraries structure. From top to bottom: colons from 1N to 4N libraries.
