# Supplementary material for: SALP, a new single-stranded DNA library preparation method especially useful for the high-throughput characterization of chromatin openness states
Source: BMC Genomics. 2018 Feb 13;19:143. doi: 10.1186/s12864-018-4530-3 (PMC5811972; doi:10.1186/s12864-018-4530-3)
Supplement: Supplementary file 6 — Figure S3. Verification of the ligation efficiency of SSA adaptors. (DOCX 21 kb) [file 12864_2018_4530_MOESM4_ESM.docx]

**File 2**

>1N-1

CAAGCAGAAGACGGCATACGAGATAAGGCCACGTGACTGGAGTTCAGACGTGTGCTCTTCCGATCTGCCAATAGATGTGTATAAGAGACAGTAGTTAGATGCTGTCATTGGATGACATTGGGCAAGCTTGTCATGTGTCTTCTGATGTCTCCCTTGTCCTTTATCAACTCACCTTCTTGCTGAACACTTTTGGAGTTTCTTGTGTGTTTATTGGCTACTGAATCTCCTTCCAACTAAATTATGTAGAGTCTAGGAAACACAGTTCTGAAATTTAATCCTGGTTCATTTGCTAGAACTCTGGATTTTTTTCCCCAAATAGTTTGGTTTCTTATACACTAATCAGGACCATTTTCCTAGTTGGAAAAAAGCAGGCACAAGGTGTGGTGGCAGAAGATCGGAAGAGCGTCGTGTAGGGAAAGAGTGTAGATCTCGGTGGTCGCCGTATCATT

>1N-2

CAAGCAGAAGACGGCATACGAGATAAGGCCACGTGACTGGAGTTCAGACGTGTGCTCTTCCGATCTGCCAATAGATGTGTATAAGAGACAGGAAGTGTTCTTGATAAAGAAGAAAGATGACTTGATTGCATTAAGGCCAGTGAGTTCCACTCTCATCCTGGAAACAAAAGAATATACTTCTAGTAGAGCAGATCTGGCAAATGATAGATGGAGAAGGCAAAACAACACTACTCATGCCTTAAGCCTGCTGCTTTCTTAAATTGAACACACAAGAAAAAAAAGATGAAAACAAGTATTTTGTTTTTACATAATTTTATTTCAAAATTTTAAGTTTCAGAAAAGAGAGTTGCATGATGTATTGTTATAATAAGAAATGCTACTTGAAAGGACTTTTGAATAAATTGAGAAAAACAAGAAAGTGATACCAAGGAGCACTGAGACAGAGATCGGAAGAGCGTCGTGTAGGGAAAGAGTGTAGATCTCGGTGGTCGCCGTATCATT

>1N-3

CAAGCAGAAGACGGCATACGAGATAAGGCCACGTGACTGGAGTTCAGACGTGTGCTCTTCCGATCTGGCCAATAGATGTGTATAAGAGACAGGATTATAATTCAATACATTAAAAATAAAATTAAAATGCAGAGTAACATTCTTCTATAGTGAAGAATGCCAGCTATTAAACACTGAACAAAGATCGAATTAGAAAAGCACAATTTAAAAAATGCACAGTTTATTAGATAAGGATAATTGATGAAATCAATGGATATTGGAAACCATGGGTGAAAGATTTTATGGGAATAAGATATTTACATAGTCCAAAATAATTCAGCCAAAATTCATCCCAGATCGGAAGAGCGTCGTGTAGGGAAAGAGTGTAGATCTCGGTGGTCGCCGTATCATT

>1N-4

CAAGCAGAAGACGGCATACGAGATAAGGCCACGTGACTGGAGTTCAGACGTGTGCTCTTCCGATCTGCCAATAGATGTGTATAAGAGACAGAGAAAACACACTGGCCAAATCTTAGCTATTTGAGGAATGTAGGGAGAAAAGCCACCTTCTCTCTCTATGTCTGAAGGTTCCCATGGCTGTCTCTTTGCCCAAGGGGCAAACTTTCCATCAGGGCATCTTCTGTGCCTCTGAGGATCATTTTCCAATTATAGGCAATGGTAGTACGTGTTTCAGTGCAGAATGAGATAGAGTTGTTTAATTTGACAATAAAGCGATGCGTCAAAAACCTCAGTCAACACAGTAAGTGTTTTCTTGTTTTCCTGCTGACCAACCTAATTCTGGTTTCATACAGGGCAGCCAGATCGGAAGAGCGTCGTGTAGGGAAAGAGTGTAGATCTCGGTGGTCGCCGTATCATT

>1N-5

CAAGCAGAAGACGGCATACGAGATAAGGCCACGTGACTGGAGTTCAGACGTGTGCTCTTCCGATCTGCCAATAGATGTGTATAAGAGACAGGGGTGGTTTGCTTTCCAAGGGGCATGGAGATGGAGATGCTACAGAACATGCCACGCTTGATACACTTCGCATCGCCCAGGTGCCCCTACTGCGTGTCATGTGACGGTGGGTGTGCCTGGTTGAGGACACCTTCTAGTCTCATGTGTGAAACACAAGCTTGTTTGTTTGACATAGTCTGTTGTGTAGTTAATGTTAGATCGGAAGAGCGTCGTGTAGGGAAAGAGTGTAGATCTCGGTGGTCGCCGTATCATT

>1N-6

CAAGCAGAAGACGGCATACGAGATAAGGCCACGTGACTGGAGTTCAGACGTGTGCTCTTCCGATCTGCCAATAGATGTGTATAAGAGACAGGTCTCACCTTCAACCACTGTGTGCTAATCCCAGCAGGCTGGGTGAGGTGTGTAGATGGTATCTCACGTGGAGATCTTGCAGGGTAAACCTTAAGATCTACTGACAAATACTGATTCCAGTTGGAAGCATTAGTACATTTTGAAATATTTAATAATTTTAACTTTTCTTAGATATGCCCCACTTGGGGACTATCTTTAAGGGCCATGAAACCGGTATGATAGTAATTCTTAAGATTTTTAAATGAAGAAAAGCAGGAGAAATGTTGGTAATAGGATCAGTCAAATATCTGCTAGTTGAAACCACCAGATGCAAATGTTTTAAGTTTCTTCCCACTGCTACTTTCCACTCTAATATAGCTTGTTGAAAGAAAATAAAATTTGATCATGCGGGCTAGTCAGATCGGAAGAGCGTCGTGTAGGGAAAGAGTGTAGATCTCGGTGGTCGCCGTATCATT

>1N-7

CAAGCAGAAGACGGCATACGAGATAAGGCCACGTGACTGGAGTTCAGACGTGTGCTCTTCCGATCTGCCAATAGATGTGTATAAGAGACAGGCCTGTAGGTTTTTTTCTTTTAACTTGTGATTTTTAAAATGAAGTAATTTAAAAATTGGGAAATTTCACATAAAAACCCAGATTTTTGGAAAAATCAGATGATCTAGCACAACTATGCTTGGATTCAACGTGATGATGATCCTGGCCACGCGAGGGGGCTGCCTGTTTCCACTGAGATATCTGCTCTCTGCCTGACAGCTGTTCCCATCAGGCCCCACAGTCTTGCATCTGCCTGCCTTCCACAGTGGCCTCACCTGTGGGCTTGCATACATCCCTGAGTTTGGAACTCATGTTCTGTTGATCATTTCTCACTTAACTAATACCCATGGGCTTCAAAGACTTCAGCAGATCGGAAGAGCGTCGTGTAGGGAAAGAGTGTAGATCTCGGTGGTCGCCGTATCATT

>1N-8

CAAGCAGAAGACGGCATACGAGATAAGGCCACGTGACTGGAGTTCAGACGTGTGCTCTTCCGATCTGCCAATAGATGTGTATAAGAGACAGTATTTGGATTCTGGGCTACCATTTTATTTCATGCATTTTGTTTTGCTGGTTTAATTTCCTTTTTCTCTTCTTTGGAGTTGACTTCTATCATTCAATTTTTCTTCTCCTCTACTTGTTTGGTTTCTATTTTTAATGTATACATTGTATGCACTATGTATACATGTATATAGTGTATATAAGCATATGTATATGTATCATGTATGTGTATATGTATATATATGCCTACAAATGAAGATTACTTAAATCTTAGCAACTAGTCTAAAATAATGAAGGCTTAGAAACTGGAAGGGAAGAGAGCTTAGTGGTAGATCGGAAGAGCGTCGTGTAGGGAAAGAGTGTAGATCTCGGTGGTCGCCGTATCATT

>1N-9

CAAGCAGAAGACGGCATACGAGATAAGGCCACGTGACTGGAGTTCAGACGTGTGCTCTTCCGATCTGCCAATAGATGTGTATAAGAGACAGGTGCATTCAACTCACCGAGTGCAACATTCCTCTTGATAGAGCAGTTTGGAAACATTGTTTCTGTAGAATCTGCAAGTGGATATATGGACCGCTTTGAGGCCTTCGTTGGAAACGGGATTTCTTCCTATAAACCCAGACAGAAGAATTCTCAGAGATTTCTTTGTGATGTGTGAATTCAACTCACAGTGTGGATCCTTCCTTTTGATAGAGCAGTTTTGAAACACCGTTTTTGTAGTATTTCCAAGCGGATATTTGGAACGCCTTGAAGCGTATGGTAGAAAAGGAAATATCTTCCCATAAAACCTAGACAGAACCAATCTCAGAAACGACTTTGTGATGTCTGCATTCAACTCACAGAGTTGAACATTTCTCTTGATAGAGCAGATCGGAAGAGCGTCGTGTAGGGAAAGAGTGTAGATCTCGGTGGTCGCCGTATCATT

>1N-10

CAAGCAGAAGACGGCATACGAGATAAGGCCACGTGACTGGAGTTCAGACGTGTGCTCTTCCGATCTGCCAATAGATGTGTATAAGAGACAGGAGAACAGTATTGAAATATGGGCATCCAGGCCAATTGCAAGAGGACAGATCTTGAGTCCTAATCCATTTGAGTTTATTACAAGGACAGAAGTGAAGTGAAAGGGAATTACTCATTTTTGTTTTTCAACATTTGCCTCTTGAATCAAGAGAGAGGGTGGAGCCTCTCTTGCTTGTGAGGCAGGGTGTTTCCACATACTCGTAACTTGAACTCTAGGAAGAAAAAGGTAGCAGGATAAATTTTACAGAAAAGGGAAGTAGAGCAGCATGCTTTGCCCAAGCACTCATCTCCTTTGATACAGTTCCTTCAGATACTTTGAAATGACTAATGCATTATATTTAAGGCCACTAGTACTAGTCATTGTGTTTTCAAGGAAATCAGAGGTATTCCCTGCTTCACTAAATGTATTTGTCATGACCAAAGATCGGAAGAGCGTCGTGTAGGGAAAGAGTGTAGATCTCGGTGGTCGCCGTATCATT

>2N-1

CAAGCAGAAGACGGCATACGAGATAAGGCCACGTGACTGGAGTTCAGACGTGTGCTCTTCCGATCTGCCAATAGATGTGTATAAGAGACAGGCTGTCAACCTGAGTCATGGGGGTGGGATGAGGGTAGGGGGCAGAGTAATGTTTTCTCTAGGTCACATACTTTGTATTCAACTTATAGCTTGAATCTTCAGATTGGCAACAAGTGCAACATTGGCAAATCTTACAATTCCCTTGCAATTCACAAGTTACAAAGCACTTTAACCAGAACATCCTCAGAACAACATACTGTAACATTGGTAGAGTTGGTATTATCATCACTTTTTAAGGAAAAGATATAGGAAGCTTAGTAAAGCTAAGCAAACTATTCAAATTCACACAGAGAGTAATTAGAAGAAAAGGATTAAAAACAGGTCTCTAGAGTTCTCTCCAAAGTACCATGGTACTCCAAAAATAAATTATTGCAGCGTTCTTTGAATATATCATCACACTTCATTTTTATAAAACATTTGGGCTATTTATATGTATGCATATACAACTGATACTTTCAAACAATTATACCATCCTTTCATAAACACTTGTACTCCAACTTTTTAATAAATGAAGTCAGGTCTAGAAAAATATACCCTTAAGTTCCACCAAAATATAGACTGCTGCACAAGATCGGAAGAGCGTCGTGTAGGGAAAGAGTGTAGATCTCGGTGGTCGCCGTATCATT

>2N-2

CAAGCAGAAGACGGCATACGAGATAAGGCCACGTGACTGGAGTTCAGACGTGTGCTCTTCCGATCTGCCAATAGATGTGTATAAGAGACAGGGTCATTTTACCTTCTCAGTTAAAATTAAACATTTATTCTTTGTGTCAATAGCACTTGAATGTATAGTTAGAATACTTATCAAATTATTGTGCTTGTTTATATATTTATTTCCCTGATTAAGAGAGGAAAAAAAGAATAACTTTCTATTTCATTTCCTCAGAATCTGCCCTGATGTTTAGCTCAAATAGATGTTAATGAGTATTTATTGAATTAAGAATGAAAAAATTTAAGCCAACAAATGTATAACTGTGTTCTCTGTCTTGTTCAAGTTGAGGAATACATAAACTAGGTTACTTTAGAGAATAAATGAGCAAAGAAAATGAGCTTTTAGTGCAGATCGGAAGAGCGTCGTGTAGGGAAAGAGTGTAGATCTCGGTGGTCGCCGTATCATT

>2N-3

CAAGCAGAAGACGGCATACGAGATAAGGCCACGTGACTGGAGTTCAGACGTGTGCTCTTCCGATCTGCCAATAGATGTGTATAAGAGACAGCTTCCGTCTCCCCCACTGGACTCTGAATTCCTTGAGGGTAGGGATTGTGCCCTTCTTCCCAGTGCCTGCCACAGAAACGGTGCCCAGTAAACACGTATTTGTGGAATTGATGAATTGGAGTTGGTCTCTGCCCTGGGTGTTTCCCATCAGTCTCGCTGTCCCGCCCTTCTGCCCTTCTGAAGCCCATAAAACAGAGTCTGCTCCCCAAGCTGGCCTGGCTCGGGTCGGGGCTCGCAGCGTCCCCTCCCCAGCAAGATCGGAAGAGCGTCGTGTAGGGAAAGAGTGTAGATCTCGGTGGTCGCCGTATCATT

>2N-4

CAAGCAGAAGACGGCATACGAGATAAGGCCACGTGACTGGAGTTCAGACGTGTGCTCTTCCGATCTGCCAATAGATGTGTATAAGAGACAGGTGTGGGAAGGTGGGTGGAAAATGAGTTTTTGTTAATATTCAAAGGCATGAAAACATTTTTACCAGTTTATGTTTTCCTGGTGCATTTAGAAATCTGTGGATCCTTGGGGATGGTGTATGCAGGCAAATAGAGAATCCAGTACTTGTGAATCTGCCTGAATCCACAGGTTTGGGAATAAGGGCAGGGACTTGAGGGTTCACAGATGTGAAGGTTGTACACAGAACTCATGCAGAGAGATACAAGATCTTTTTGTTCCCCCTTTGATTAGAAAGAATAGGACATGAAAGTACTTAATTGTCAACTTCGTCTTCACCATAAGCCCAGTATTGATGCAAAAATGATAATAATAATGAGAACAAGCATTTATTGAGTATTGGGTATTCTAATTGCTTAAATCAACTCATGTAATTCTCACAAGATCGGAAGAGCGTCGTGTAGGGAAAGAGTGTAGATCTCGGTGGTCGCCGTATCATT

>2N-5

CAAGCAGAAGACGGCATACGAGATAAGGCCACGTGACTGGAGTTCAGACGTGTGCTCTTCCGATCTGCCAATAGATGTGTATAAGAGACAGGCCTAACTGAATTAGGGGAAAGAAAATACCCAACTCCGACTCCCTCCAGCCTTCCACGTGGGAGAAGAAAAATACCTAATTCCAGTTCATCCTAGCCATCCTGTCCCATGCAAGGGGAAAGAAAGTAACCTGAGAAGCACTTGTGAAGTTCACAGTCAAAAGGCATAGGCTCGCTAAAAGACAGACTGAATCACAGGACTATAGAGTGTTTCTCCTCTTCCCATACCTTGCTAGTACAATATTAAAGCCTTATTTGTGGCTGTTTCTTTTAACTGGTACATCATGTTCAGCTATAATGGATATGATGATTATAAAAAAAATACGAGGTGTACTAAAAGGCAAGAACAAACAATAAAAAAAAAACCCCACAATTTGAGGAGACAGAACAAGCAGCAGAACCAGTCTTGGATGAGATGAAAAAGATCGGAAGAGCGTCGTGTAGGGAAAGAGTGTAGATCTCGGTGGTCGCCGTATCATT

>2N-6

CAAGCAGAAGACGGCATACGAGATAAGGCCACGTGACTGGAGTTCAGACGTGTGCTCTTCCGATCTGCCAATAGATGTGTATAAGAGACAGATACATTAGAAATGGAAAGCTATGGAAGATTCCACAAAGAGAAATAGATAATATTTTGAAACCTTACTCTAAGGAATATGACAATGTGGGATATCCTCCCTGCCCTCAACCCTCCCCCTTGTTCCCATTCCATTTCTTCTCCTTTAGAGCTTTGAAGAAAACGCATTTGGTATTTAGTAATCAGGATTAAACAATATAAGCACATCACACCTCTTAGCTCACTTTTTCTGATAACTGCACAGAAACAAGACTCTGTCATAAGATCGGAAGAGCGTCGTGTAGGGAAAGAGTGTAGATCTCGGTGGTCGCCGTATCATT

>2N-7 CAAGCAGAAGACGGCATACGAGATAAGGCCACGTGACTGGAGTTCAGACGTGTGCTCTTCCGATCTGCCAATAGATGTGTATAAGAGACAGGAACACTGAACTATGGGAGGGTACACCCAACATTGCTGGAGACACCATGCCCTTCACAGGGTCCTCCCAAAGCGCTCCAGACACCAGCACCCTGGATAAAGAACCTGCCACTTTGTCCCAGGGGCTGAGGCTTCTCTCCAGTCTCCTGCGTCTACCCCATTTTCAAGCCCTCTTGCTTTGGTCTCATGTGCCCACACTTTCAACCCAAACTGTGCCTTTCTGGCCAGTCTCTATGGATGAATACCTCAGCTGAACTGTCTACCTGGCTTTCCATAAGATCATCTTTGGTTCCAGGATCTACAATAAAAGCACCAGACCTGATCTATCCCAGTCTGCTCTGACCTCATCAAGATCCGGAAGAGCGTCGTGTAGGGAAAGAGTGTAGATCTCGGTGGTCGCCGTATCATT

>2N-8

CAAGCAGAAGACGGCATACGAGATAAGGCCACGTGACTGGAGTTCAGACGTGTGCTCTTCCGATCTGCCAATAGATGTGTATAAGAGACAGGAGTGTACATCTTAAATAACCAATTTGTAATAAATTTAATCAGCTAGAAAACAAGTGTAACTTTTGCAACTTTTGAAAAACACACATCTCTGGGCATCAATGAAAACTCTTCCCTCTACAGTAAGCCTAATGAAGTGCAACTAAAAATAACAGTCATCAACTGTGTTTTAAAGGCAGTATTTCAACATAATCAAATGTGTCAAATATTCATCCTTACAGCTTCTTATGCTGTGGGTTATAAGTAAGTTTCATTTCTTGGGAATGACTGAACATAACCCACCTGGGGCTCTGCCATCTGTGAATTACTTATATGTGAACACTCTTTAAGAGATGGAAATTTTGATTGTTTTTTCTTCCTGTAGATCGGAAGAGCGTCGTGTAGGGAAAGAGTGTAGATCTCGGTGGTCGCCGTATCAAT

>2N-9

CAAGCAGAAGACGGCATACGAGATAAGGCCACGTGACTGGAGTTCAGACGTGTGCTCTTCCGATCTGCCAATAGATGTGTATAAGAGACAGCTTTTGGATTTTTTTAAATATTTTATTTTTTATAATTCAAGCAAAGATCAAAAAAATATTAAAAAAATAATTGCTCGACACTATCAATACTTTAGTGTTAAAGAAACTCTAAAAAAATTTGATAAAAATTCAAATAAAGTTGGAATTGTATGACATGTTCAAGGTTCAGGTAAATCCCTTACAATGGTAATGTTGACTAAGTTGTTAAGAACAATTGAAAAGAATTTAACAGTTATTGTGGTAACTGATAGAATTGATCTTCAAGATCAATTGAACAACACTTTTAATAACTTTCATAAATATATTGGTAGATCGGAAGAGCGTCGTGTAGGGAAAGAGTGTAGATCTCGGTGGTCGCCGTATCATT

>2N-10

CAAGCAGAAGACGGCATACGAGATAAGGCCACGTGACTGGAGTTCAGACGTGTGCTCTTCCGATCTGCCAATAGATGTGTATAAGAGACAGTTACAATCCTGCTCTTGTGGCTGGGATCAGGAATATGAGCAAAGGAGGCCACGAGAAGGAATCACATGTGTAAAAGGTGGCTTGAATTATTATTTTTTAAAATATCATGGAGGCTTATTATGAGACAAATCATCAAGATAGCTGACAATAGATATAATGTTCAGCCACTTCAGGTCTTTGCCTTTTCTGTCCACACTATATTTATTTGCACACAAATACCACCAATGCCACTACCACTGTCACTAGTCCCAGTTAGCCTTATTGTTCTCCATAGCATTGAGCACAACTAGTCATTCCACGTATTTTACCTTTTTATTTTCTTTATCTTATGCCTCATTCTACCAGCATGGAAACTCAAATAAATCATTGATTTGTTTATTTTTCATTTGTGCACCCTCAATACCTAAAACAGTACGTGGCACAGGGCAGGGATTCAAAAAGTGTTTGTTGACTGAATGGTCAGATCTATTATTTTTTGAGACATTCTCTCTAGAAAGATCGGAAGAGCGTCGTGTAGGGAAAGAGTGTAGATCTCGGTGGTCGCCGTATCATT

>3N-1

CAAGCAGAAGACGGCATACGAGATAAGGCCACGTGACTGGAGTTCAGACGTGTGCTCTTCCGATCTGCCAATAGATGTGTATAAGAGACAGGGCATATACATTCTAAATATTCTAATAAAAACTTTTAGAGATTACCAAACAAGTACTTTTATTTTTCCATTTAAAATAGGATAGAATGGATAGTCAAGATCTATCCAGTCTTCTGTTTCACTTTGGGAAAATCCCCATTTGCCTCATATTAGTTTGTAAACATCTCACGTTTTTCCCAAGTCTCAGTAGTTTTAAGTGCAAATGTTACCACCAACAATCACATTTTTAACTATATCTATTTCGTCCCTAAAAAAACTGGTGTTTCTCAGATCGGAAGAGCGTCGTGTAGGGAAAGAGTGTAGATCTCGGTGGTCGCCGTATCATT

>3N-2

CAAGCAGAAGACGGCATACGAGATAAGGCCACGTGACTGGAGTTCAGACGTGTGCTCTTCCGATCTGCCAATAGATGTGTATAAGAGACAGGGGAGAGGATCAGGAAAAATAACTAATGGGTACTAGGCTTAATACCTGGGTGATAAAATAATCTGTACAACAAACCCCCATGACACAAGTTTACCTATGAAACAAACCTGCACTTGTACCCCTGAACTTAAAAGTTAAAAAAAGTGCATATATACAATGAACAACTATTCAGCCAAAAAAAATGAATGAGATCCTGTCATTTCCAATAGCATGAAAGGAACTGAAAGACATTAAGTGAAGTAAGTCAGGCACAGAAAGACAAACTTTGCATGTTCTCACATATTCGTGAGAGCTAAAAAATTAAAACAATTGAAATCATGCAGATAGAGAGTAGAATTATGGTTACCAGAGGCTGGGAAAGATCGGAAGAGCGTCGTGTAGGGAAAGAGTGTAGATCTCGGTGGTCGCCGTATCATT

>3N-3

CAAGCAGAAGACGGCATACGAGATAAGGCCACGTGACTGGAGTTCAGACGTGTGCTCTTCCGATCTGCCAATAGATGTGTATAAGAGACAGCTATATAAACATCTGACTTCTCAACATAAATAGTGGAAATTAACAGACACTGGAATGATAATTCAAAGTGCTGAAGATGAAAAATCAAGAATTCTATATTTAATGAAATTATCTTTTTAAAATGGAGGCCAAAAATACATTTTTCAGATCAACAAAATCTAAGATAATTTGATTGTAACAAATTTATACTTCAGGATGGACAAGAAGTTCTGTCAGCTGATGAGAAATGATGCCAGATGGTAACTCAGATATACAAGAAATACTGAAATAGATCGGAAGAGCGTCGTGTAGGGAAAGAGTGTAGATCTCGGTGGTCGCCGTATCATT

>3N-4

CAAGCAGAAGACGGCATACGAGATAAGGCCACGTGACTGGAGTTCAGACGTGTGCTCTTCCGATCTGCCAATAGATGTGTATAAGAGACAGACCATATGGAATGAGATGATAAAATTATTGGATTTAACAGAACAGTGTGGGAGGTAAAAAAAAAAATCAAGAATTTTATTGGCACAAATTACTGGTTTCTATCCCCTATTTTCTCAACTATAATTCTTTTACATTCCTTCATTCTTTCCTCTGGACCCAATCATAATGTAATTCCTAAATCTAGTGGTTTTTGTCAGCATTCATCCTACTTGAACTTTCTTACAGTGTTTGACAAACTACATTCTAATTCTGGAGCTCTGTCTTTTCACATCACTCTATCTCAGCTTCCAGAATACTAGATCGGAAGAGCGTCGTGTAGGGAAAGAGTGTAGATCTCGGTGGTCGCCGTATCATT

>3N-5

CAAGCAGAAGACGGCATACGAGATAAGGCCACGTGACTGGAGTTCAGACGTGTGCTCTTCCGATCTGCCAATAGATGTGTATAAGAGACAGATATAATGCAGTGCCTCAGACATAATTAAAAACCTTATAGAACTGAATTGAATGTCCATGCCATTTATGGCTGGATGATGGCAAGAAAACAGATTGTAGGAAGGAAAAATCTTGCCATCATGTCCAGTTGGGATGCCGAAATGCTTCAGACTTTTTTTTTTTTTTTTTTAAGAAAAAGAATTTGTGTCTACTGGACAGGAAATTAATTCATTTCCAGAACAAGTTTTTTCTTAAAACACGCTAAGGTCAAACTTCCCATAATGCCTACTGTCATGGTGGTTGTCTATGATTGGTATAGGCACATCCCAAAGCAATAAATTCATCTCCTAAAGGACCACTGTGCTAATGCTTGCCTGACAACCTGCTTCAAGAAAATGTGTCTAACTCCATTACTAACATTGAGTCATCACTGTCCAATTCTTTCTCTTTAATGTTTAAGAGTAAAGATCGGAAGAGCGTCGTGTAGGGAAAGAGTGTAGATCTCGGTGGTCGCCGTATCATT

>3N-6

CAAGCAGAAGACGGCATACGAGATAAGGCCACGTGACTGGAGTTCAGACGTGTGCTCTTCCGATCTGCCAATAGATGTGTATAAGAGACAGGAATTGAATCATCACCAAATTGAGTCGAATGGAATCATCAAATGGAGTGAAATGGAATCATCATCGAAGGGAATGGAATAGAATCATCGAATTGACTCGAAAGAATAATCATCGAAGGGAACGGAAAGGAATTATCCAATGGAATACAAGAGAAACATCATCAAATGGAATCGAATGGAATCATCATCGAAAGGAATCCAATGGAATAATCATCAAATGGATTCATACGGAATGATAATCGAATGGAATTCAAAGGAATCATCATCGAAGGGAATCGAATGCAACAATCGAATGGAATCTAATGGAATCATCATCGAATGGAATCGACCGGAATCATCGAATGGAAGAGAAGAGAATCATCATTGAATGGAATTGAATGGAATCGTCAATGAATGGAATCGAATGGAATAATCAGAGAATAGATCGGAAGAGCGTCGTGTAGGGAAAGAGTGTAGATCTCGGTGGTCGCCGTATCATT

>3N-7

CAAGCAGAAGACGGCATACGAGATAAGGCCACGTGACTGGAGTTCAGACGTGTGCTCTTCCGATCTGCCAATAGATGTGTATAAGAGACAGATACAGGAGAACTCCAAACCAAGAAAGGTTTTTTTTTTTTTTTTGGCCAGACTCTGAAAAAGTAGGCTGTAATATATATTTTTAAAAAGTCTATACCCATGAAGGACCGTGTGAGGAGATGCTATCTTATATAGAATAGGGCTGAGGCTTATTGAGGCTTTGCCAAGATTTCAGAGTAAATCTTATTCACTTTGAATAAGAAATTTGTCTTATGAGAAAACTATTGGCTTGAAATGTGGTGAATACAAGGGCTGAGGGAGACTCCAGTGGGTTTGTACCTATTCTCAGCCTTACCCAGGAGCTGGCTGAAATGGGTTAGTTGATGGAAAAATCTCTTTGTGTGTGTAGATCGGAAGAGCGTCGTGTAGGGAAAGAGTGTAGATCTCGGTGGTCGCCGTATCATT

>3N-8

CAAGCAGAAGACGGCATACGAGATAAGGCCACGTGACTGGAGTTCAGACGTGTGCTCTTCCGATCTGCCAATAGATGTGTATAAGAGACAGATCATCAAGTTCAAACTGCTTATCCTAGCAATGCAAACTGACAGCATATGCACATACATAATCAAATGGGAAATAATAATAGTATGTCTCGGCGGACTGCCTAACACAAGAAAGTCAACGGCCCAAGGGATATGTCAAGAATAATTTCTTTGCATGCATTGCTCTGATGCTATGCTTTGACACAGGATTATTTCATCAGTGGAGAGTGTATGAAACTCTTAGGCAACAAACTGGATTTTCCTTTTCAGCCAGATCGGAAGAGCGTCGTGTAGGGAAAGAGTGTAGATCTCGGTGGTCGCCGTATCATT

>3N-9

CAAGCAGAAGACGGCATACGAGATAAGGCCACGTGACTGGAGTTCAGACGTGTGCTCTTCCGATCTGCCAATAGATGTGTATAAGAGACAGGCAGTAGATAGTGAAGTTTCCTTTTTTCAGTAGAACTGAAACAATCAAAGAAATGTTATTTTAGTAAATGTTGATCTCTTTTCATTCTTTCTCCCTGGACATTCTCTGAAATCTCTGTCAATGGAATTTGTTTACTCCAAATCACATCTTTGTTGCCTTTGAGTATTACCATGTTTGAATGTTTACCACTCAAATCCAGCATAAAAGTGTCTTCTTTTTTAGGTAAGATCAGGCAAAGAGGTACTGAATGAATAACACTTGATTGGGAATGGTAAATAACCATGCAATTAAACTGTAAACACTGTGTGTGGTGATTTTAATGTAATTTGAGGACTTGTAAATTATATGGTCATAAAATGGCACTTGGGCTTATGCTTTACAAAAATATCCATGTTTGTATGAGATTAATTAGCGCAGTTTGCATAAAGACATTGAGTAAAGCACTTTCTGAACATTCTCATTTGTAAGGTTTTCTTATTTATAAGGCTTTCTTTTTATTTCTTTCTGTGGTCTTGAAGAAATTATTATCATTGTCCACAGATCGGAAGAGCGTCGTGTAGGGAAAGAGTGTAGATCTCGGTGGTCGCCGTATCATT

>3N-10

CAAGCAGAAGACGGCATACGAGATAAGGCCACGTGACTGGAGTTCAGACGTGTGCTCTTCCGATCTGCCAATAGATGTGTATAAGAGACAGGTGCACCCTCACAGTTCAAACCTGTGTTGTTCAAGGGTCAACTGTATATCCAAGTTCATACATATCGTAAATGGTAGAACTAAGATGCAATTTCAGATCCAAATTCAGATTTTCAAATTCAGTTTCCAAGTCATATGATGACACTACTTAGAAAATCAAAATTAGTTTCCAGCTTTTACAAATCAAGCTGCTAGTAGTAATTCTAATACCATTATATGATTATTAATAATGCCACCACATTGATGGCTCAGCTGAGGACTAGAAAATAAGTCTTTAACAAAATTTCCTATTTGTATTTTATTTTTCTTGCAATGATGCACAGCTGAGAACAGAAAATAAGTCTTAACACTCTCCCAAAGATGGCAAGATGCACAGTCACCATGTCTAAAGATCGGAAGAGCGTCGTGTAGGGAAAGAGTGTAGATCTCGGTGGTCGCCGTATCATT

>4N-1

CAAGCAGAAGACGGCATACGAGATAAGGCCACGTGACTGGAGTTCAGACGTGTGCTCTTCCGATCTGCCAATAGATGTGTATAAGAGACAGCTTTCAATGTTCACTTCAACACTTCCGGGTATCAATCTTTGTGTATAGGAAGATCCTTAACACACTTCCTATACACAAAGCTCTGGCTCAGAGTCAACTTCCCCAGAAACAGAGAACCTGACTTCAAACAATCCCTTATTAAAACATAAAAGGTATCGTAAGTTTAGAAATCAGAAAGGCCATGAGTATAAATAGCTAAAATATGAATGCAGCAGAAAATACCTTCCTTAGAACATTGTTTTAGAAGTGGCAAACTAGGAAACTTTGAAAAGAGGTCAGTATGAAACTGTGATTTTTTTAAAAAAGATTTCATTTTGACTTAGTTTTAAGGGTGTTTCAGCCTGCAGTTATTTCAGAAGATCGGAAGAGCGTCGTGTAGGGAAAGAGTGTAGATCTCGGTGGTCGCCGTATCATT

>4N-2

CAAGCAGAAGACGGCATACGAGATAAGGCCACGTGACTGGAGTTCAGACGTGTGCTCTTCCGATCTGCCAATAGATGTGTATAAGAGACAGGTTCAGTTTCCAATCCTGTGGTTATCTTCTTTGCAATGTTGCAGCAGTTTTCAGAAGTTTAAAAGCTTTAATAATTGAAATCTCTGATATGCTTTATAGCAATGTACACGCATATGCTTTTATTCTGTAATAATTTACTGACAAAATTTACCACCTGACTGCTCGAAGTTCTTTCAGACTTAGGAGATGTTTTTCCAGCAGCTCAAGAAATGCTTTCTTGGGAGGACTTCCCATGCTCCAGGGACTTTACACGCCTCATTTCTCTTAATTCTCACAAGCAGCCAAAGAGATGGGTGATACCAAGATCGGAAGAGCGTCGTGTAGGGAAAGAGTGTAGATCTCGGTGGTCGCCGTATCATT

>4N-3

CAAGCAGAAGACGGCATACGAGATAAGGCCACGTGACTGGAGTTCAGACGTGTGCTCTTCCGTGCCAATAGATGTGTATAAGAGACAGGCCAATAGATGTGTATAAGAGACAGTGTGTACATATGTAACAAACCTGCACGTTGTGCACATGTACCCTAGAACTTAAAGTATAGTAAATAAAAAAAAGGAAAAAAATTGCTCACAAGACTGTGGAGAAAAAAGAATGCTTATATCATGTTGGTAGGACTGTAAATTAGTTCAGCCATTGTGGAAAGGAGTTTGATGATTTCTTAAAGAACTTAAAACAGATCGGAAGAGCGTCGTGTAGGGAAAGAGTGTAGATCTCGGTGGTCGCCGTATCATT

>4N-4

CAAGCAGAAGACGGCATACGAGATAAGGCCACGTGACTGGAGTTCAGACGTGTGCTCTTCCGATCTGCCAATAGATGTGTATAAGAGACAGCTTAGATAAATATCTACAAAACATAATTTATTAAAACTGACTTAAGCAAAAAACCAAAAGGTAAAACTTGAATAATGCTATAATTATTAAAGAAAAATTATTCTCACACACACACAAAAAGTACCAAGCCCCCATGGGTTTACAGGTGAGGTGAAATTTTCAAGGACCAGATCATCTAGACAAAAGAAATTCTTCCGGACAAAAGAAAAATTCTTCCAGACAAAAGAAAATGAGGGATTACTCCCTAACTCCTCTTATAAGGGGAGATGTTAAAGGAGAATGGACAGATCGGAAGAGCGTCGTGTAGGGAAAGAGTGTAGATCTCGGTGGTCGCCGTATCATT

>4N-5

CAAGCAGAAGACGGCATACGAGATAAGGCCACGTGACTGGAGTTCAGACGTGTGCTCTTCCGATCTGCCAATAGATGTGTATAAGAGACAGGGCCACCAGAGATATTTAAAAATCAGAAAATCAGAAAAAATCAGAGCCATCATTTTCAAAAATTAGTCTTCTAGCAGGGAATGAACTGAAGAAGAAAGGGGCAAAAGGTATGGAAAGTAATTAGGACAGAGCTTTTAGGGCTCCATATGTCATGCTTTTCACAATAACATGCAGAATCCACATCCTCATTTAGGTGTGATAATATCATTACTCTCTTCTCTCTCCAATGTCTACTTAGAAGTAGCGCAACAGTAAGTCTTTTTTCTGGAGATGGTCATGGCTTATACCTGTAGTCCTGGTGAAATTAATAATAGTGCCCCCATTCACTCTCGAAAGTATTCTGGTTTGGACAAGATCGGAAGAGCGTCGTGTAGGGAAAGAGTGTAGATCTCGGTGGTCGCCGTATCATT

>4N-6

CAAGCAGAAGACGGCATACGAGATAAGGCCACGTGACTGGAGTTCAGACGTGTGCTCTTCCGATCTGCCAATAGATGTGTATAAGAGACAGGAACCAGTGAGCTTGAAGATAGGACATTTGAAATTATCCAGTCAAATAATAAAAAAAGAATGAAGAAAGCCTCCAGGAATTATGGGATACCATCAAGAGACCCAACATTCACATAATAAAAGTTGCTGAAGGAGAAAAGAGAGAAAAAGAGCCAGAAAGATCGGAAGAGCGTCGTGTAGGGAAAGAGTGTAGATCTCGGTGGTCGCCGTATCATT

>4N-7

CAAGCAGAAGACGGCATACGAGATAAGGCCACGTGACTGGAGTTCAGACGTGTGCTCTTCCGATCTGCCAATAGATGTGTATAAGAGACAGACATGATACAGTGGAAGGAATACTAGAGTAGGGGTCAGGAAAATCAAATACTGTGAAGTGTGGAAGTGCCTGGCACAGAGTAGAAACTTAATCAATGTAAATCCCTCATCCCCTCCCATTGTGCATCATGAGCTTCCCAACACAGCCCATAAAATCTCCAAGTTGTAATGCTGAAAGAAGGGCCACAACCTTGTCAATGACGCAAAAGAGCTTTCATCAGACTGTGCATAATTTAGAATGTGAATCTCTGAGAAATGAGAGCTGATGAGAGCAGACTTTAGTAATCCCCTAAACTCTCAATCATCTGTGTTTTGGTAAAACAGGAGCACTAGCAGATCGGAAGAGCGTCGTGTAGGGAAAGAGTGTAGATCTCGGTGGTCGCCGTATCATT

>4N-8

CAAGCAGAAGACGGCATACGAGATAAGGCCACGTGACTGGAGTTCAGACGTGTGCTCTTCCGATCTGCCAATAGATGTGTATAAGAGACAGGGGTAAGGAATGGTGCTGAAAGCTTTTTCTCAGTGTTCCAGCTCCACCATGAGCTTTATTATGTACCTGTCCCAGAGGAAGTCTCATGCCTCCTTCTGTCCCTTCCTGGTGGTAGGCTGCTGTTGCTTGTTTCTGAGGGTTGTATTTATGGCATGGGTAGAGAGGAGGAGGTTTTCTTAGGGAATAATACTTGAATTAACTCATATTTCCACATATTTTCTTGCTTAAAAAGGTATTTTAATGATCCAAATAAGTGTTTTGACAAGTTTTCATTTATAGCTACCTCATTGAATTATTGGACTAGTAACTTTAAGAAAGCAAAAATAAGTAGTGATTTTAGACATAATTTTTTTTTGGAATGAAGTACTGGCTCCTGGTAATTGTTGTTTACTCTACAGAGCCTATGAAATCACACATAATTGATTCAATAATATTTTATGGAAACTTGCCAGAAGTCGATGTCAAAAGAACCCATCTCTAGATTACAGAATCAAACGCCCTTTTTTTTTTAACCTGGAATAATAATTTCTCTTCTATAATTTTCTATATCTTCCTCACATTCTCTGGGGTTTAAAGTGGTTTACATAATCACAAGATCGGAAGAGCGTCGTGTAGGGAAAGAGTGTAGATCTCGGTGGTCGCCGTATCATT

>4N-9

CAAGCAGAAGACGGCATACGAGATAAGGCCACGTGACTGGAGTTCAGACGTGTGCTCTTCCGATCTGCCAATAGATGTGTATAAGAGACAGGTTCAGTTTCCAATCCTGTGGTTATCTTCTTTGCAATGTTGCAGCAGTTTTCAGAAGTTTAAAAGCTTTAATAATTGAAATCTCTGATATGCTTTATAGCAATGTACACGCATATGCTTTTATTCTGTAATAATTTACTGACAAAATTTACCACCTGACTGCTCGAAGTTCTTTCAGACTTAGGAGATGTTTTTCCAGCAGCTCAAGAAATGCTTTCTTGGGAGGACTTCCCATGCTCCAGGGACTTTACACGCCTCATTTCTCTTAATTCTCACAAGCAGCCAAAGAGATGGGTGATACCAAGATCGGAAGAGCGTCGTGTAGGGAAAGAGTGTAGATCTCGGTGGTCGCCGTATCATT

>4N-10

CAAGCAGAAGACGGCATACGAGATAAGGCCACGTGACTGGAGTTCAGACGTGTGCTCTTCCGATCTGCCAATAGATGTGTATAAGAGACAGGTAGCCAATTGATTTTTGACCAGGGAACCACATTTATTCAGTGGGGAAAGATAGTTCACCAAATGGTGCTAGATTTCTGCATACAAAAGAACAAAGTTAGACCCCTACCTTACACCATATACAAAAATCAACTCAAAATTGAAAAACAACCTAAATATAAGAGTTAAAATACCAAGACTCTTAGAAGAAAACACAGGGGTAAATCTTTATGACCTTGGATTTAACAGTGGATTCTTAGATGTGTCACCAAAAGCACAAGCAACAAAAGAAAAAATAGATAAATTTGACTTCATCAGACTTTAAACTAGATCGGAAGAGCGTCGTGTAGGGAAAGAGTGTAGATCTCGGTGGTCGCCGTATCATT
