# Supplementary material for: SALP, a new single-stranded DNA library preparation method especially useful for the high-throughput characterization of chromatin openness states
Source: BMC Genomics. 2018 Feb 13;19:143. doi: 10.1186/s12864-018-4530-3 (PMC5811972; doi:10.1186/s12864-018-4530-3)
Supplement: Supplementary file 7 — Table S3. Reads from a lane of Illumina Hiseq X Ten sequencing. (DOCX 68 kb) [file 12864_2018_4530_MOESM5_ESM.docx]

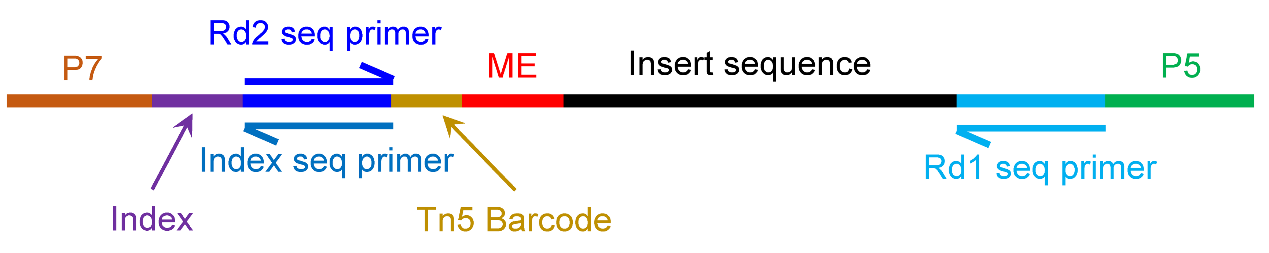


**Fig. S2. The structure of SALP library.** The SALP sequencing library structure compatible with Illumina sequencing platform was illustrated. And the name of the each element were shown in the figure.
