# Supplementary material for: SALP, a new single-stranded DNA library preparation method especially useful for the high-throughput characterization of chromatin openness states
Source: BMC Genomics. 2018 Feb 13;19:143. doi: 10.1186/s12864-018-4530-3 (PMC5811972; doi:10.1186/s12864-018-4530-3)
Supplement: Supplementary file 8 — Figure S4. Comparison of fold enrichment of two types of GM12878 SALP-seq peaks. (DOCX 187 kb) [file 12864_2018_4530_MOESM6_ESM.docx]

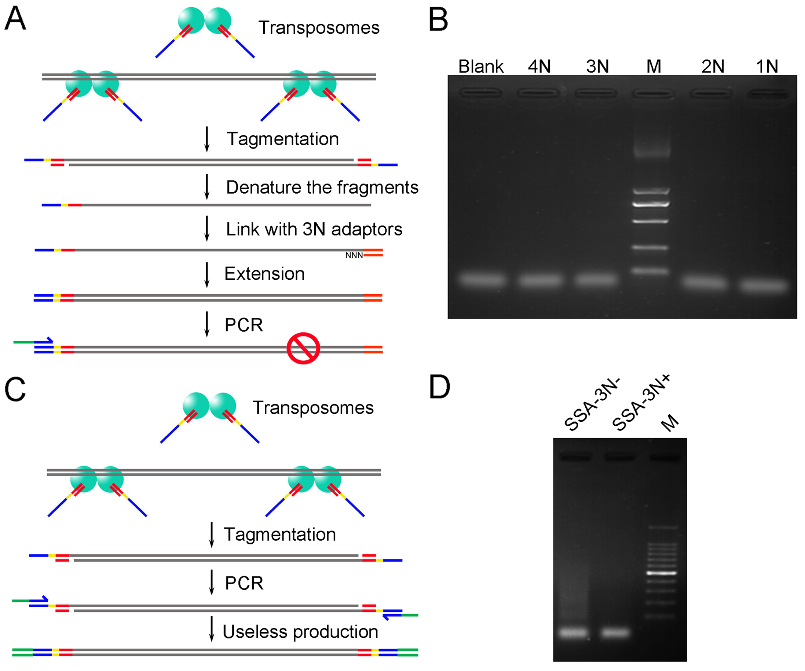


**Fig. s3. Verification of the ligation efficiency of SSA adaptors.** (A) Schematic of tagmentated-based SALP with successful SSA adaptor ligation. The fragments ligated with SSA-3N were amplified with PCR by using a single primer annealing to Tn5 adaptors for 35 cycles to check the ligation efficiency. (B) The agarose gel showing successful ligation of different SSA adaptors. Four SALP reactions were performed, each contained one of SSAs (1N‒4N). After elongation, the products were amplified with PCR by using a single primer annealing to Tn5 adaptor. A PCR negative control was also performed. The PCR products were run with agarose gel. (C) Schematic of a useless PCR amplification with a single primer annealing to Tn5 adaptors. (D) The agarose gel showing ligation efficiency of SSA adaptors. Two SALP reactions were performed. One was added with SSA-3N in the SSA ligation step (SSA-3N+). The other was not added with SSA-3N in the SSA ligation step (SSA-3N-). After elongation, the products were amplified with PCR by using a single primer annealing to Tn5 adaptor. The PCR products were run with agarose gel. It can be seen that the SSA ligation prevented the single-primer PCR amplification, indicating the successful and high-efficiency SSA ligation in SALP method (B and D).
