# Supplementary material for: SALP, a new single-stranded DNA library preparation method especially useful for the high-throughput characterization of chromatin openness states
Source: BMC Genomics. 2018 Feb 13;19:143. doi: 10.1186/s12864-018-4530-3 (PMC5811972; doi:10.1186/s12864-018-4530-3)
Supplement: Supplementary file 9 — Figure S5. Construction of NGS library of gDNAs sheared by sonication and restriction endonuclease digestion with SALP method. (DOCX 15 kb) [file 12864_2018_4530_MOESM7_ESM.docx]

**Table S3. Reads from a lane of Illumina Hiseq X Ten sequencing.**

| **Pooled DNA samples** | **Barcode** | **Index** | **NGS library** | **Reads number** | **Mappable reads number** |
| --- | --- | --- | --- | --- | --- |
| GM12878 10^5^ cells | TAGCTT | AGTCAA | **NGS-L1** | 12845281 | 10760571 |
| HepG2 10^5^ cells | CTTGTA |  |  | 17474263 | 12329768 |
| HeLa 10^5^ cells | GCCAAT |  |  | 19120478 | 16439189 |
| 293T 10^5^ cells | TGACCA |  |  | 10647259 | 8828634 |
| HepG2 5×10^4^ cells | ATCACG | AGTCAA | **NGS-L2** | 27245787 | 20946874 |
| HepG2 1×10^4^ cells | ACTTGA |  |  | 399898 | 212125 |
| HepG2 5×10^3^ cells | CGATGT |  |  | 134725 | 95232 |
| HepG2 2.5×10^3^ cells | ACAGTG |  |  | 116644 | 86011 |
| HepG2 5×10^2^ cells | CAGATC |  |  | 26425 | 7356 |
| HindIII-digested HepG2 gDNA | - | ATGTCA | **NGS-L3** | 32944329 | 14140187 |
| Sonicated HepG2 gDNA | - | AGTTCC | **NGS-L4** | 51687071 | 11356768 |
