# Supplementary material for: SALP, a new single-stranded DNA library preparation method especially useful for the high-throughput characterization of chromatin openness states
Source: BMC Genomics. 2018 Feb 13;19:143. doi: 10.1186/s12864-018-4530-3 (PMC5811972; doi:10.1186/s12864-018-4530-3)
Supplement: Supplementary file 10 — Figure S6. Comparison of the distribution of Hind III digestion library reads density and Hind III restriction sites through the whole genome. (DOCX 45 kb) [file 12864_2018_4530_MOESM8_ESM.docx]

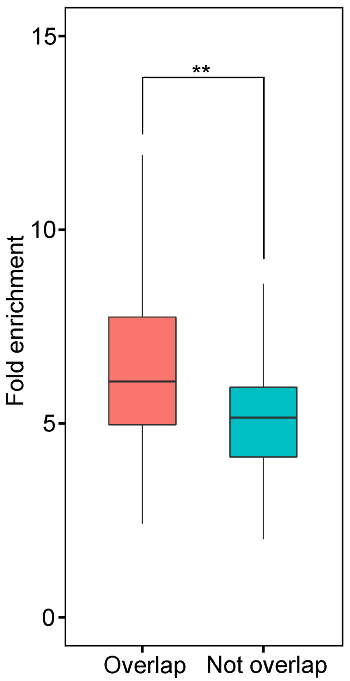


**Fig. S4. The comparison of fold enrichment between two types of GM12878 SALP-seq peaks.** Overlap, SALP-seq peaks overlapped with GM12878 H3K27Ac peaks; Not overlap, SALP-seq peaks not overlapped with GM12878 H3K27Ac peaks.
