# Supplementary material for: SALP, a new single-stranded DNA library preparation method especially useful for the high-throughput characterization of chromatin openness states
Source: BMC Genomics. 2018 Feb 13;19:143. doi: 10.1186/s12864-018-4530-3 (PMC5811972; doi:10.1186/s12864-018-4530-3)
Supplement: Supplementary file 11 — Figure S7. Reads distribution of sonication library. (DOCX 295 kb) [file 12864_2018_4530_MOESM9_ESM.docx]

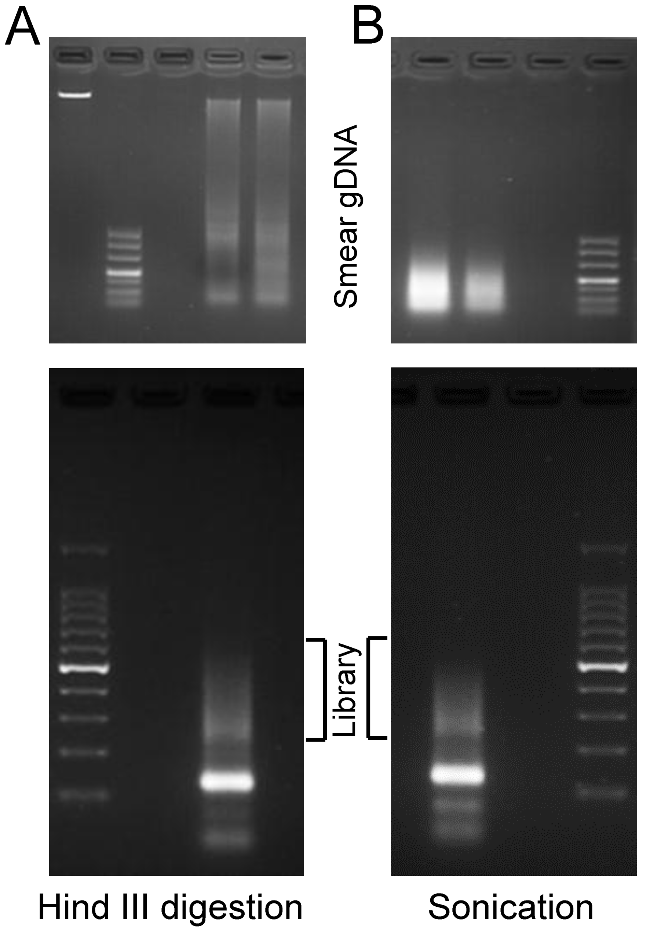


**Fig. S5.** Construction of NGS library of gDNAs sheared by sonication and restriction endonuclease digestion with SALP method**.** (**A**) The library constructed with the Hind III-digested HepG2 genomic DNA. The HepG2 genomic DNA digested by Hind III were shown on the top, and constructed library were shown on the bottom. (**B**) The library constructed with the sonicated HepG2 genomic DNA. The HepG2 genomic DNA sheared with sonication was shown on the top, and the library constructed with sonicated DNA was shown on the bottom.
